# Epithelium-Intrinsic MicroRNAs Contribute to Mucosal Immune Homeostasis by Promoting M-Cell Maturation

**DOI:** 10.1371/journal.pone.0150379

**Published:** 2016-03-01

**Authors:** Gaku Nakato, Koji Hase, Takao Sato, Shunsuke Kimura, Sayuri Sakakibara, Machiko Sugiyama, Yuuki Obata, Misaho Hanazato, Toshihiko Iwanaga, Hiroshi Ohno

**Affiliations:** 1 Laboratory for Intestinal Ecosystem, RCAI, RIKEN Center for Integrative Medical Sciences (IMS-RCAI), Kanagawa, Japan; 2 Laboratory for Immunobiology, Graduate School of Medical Life Science, Yokohama City University, Kanagawa, Japan; 3 Division of Mucosal Barriology, International Research and Development Center for Mucosal Vaccines, The Institute of Medical Science, The University of Tokyo, Tokyo, Japan; 4 PRESTO, Japan Science and Technology Agency, Tokyo, Japan; 5 Laboratory of Histology and Cytology, Graduate School of Medicine, Hokkaido University, Hokkaido, Japan; 6 Graduate School of Medical and Pharmaceutical Sciences, Chiba University, Chiba, Japan; The University of Tokyo, JAPAN

## Abstract

M cells in the follicle-associated epithelium (FAE) of Peyer’s patches (PPs) serve as a main portal for external antigens and function as a sentinel in mucosal immune responses. The scarcity of these cells has hampered identification of M cell-specific molecules. Recent efforts have begun to provide insight into antigen transcytosis and differentiation of M cells; however, the molecular mechanisms underlying these processes are not fully elucidated. Small non-coding RNAs including microRNA (miRNA) have been reported to regulate gene expression and control various biological processes such as cellular differentiation and function. To evaluate the expression of miRNAs in FAE, including M cells, we previously performed microarray analysis comparing intestinal villous epithelium (VE) and PP FAE. Here we confirmed FAE specific miRNA expression levels by quantitative PCR. To gain insight into miRNA function, we generated mice with intestinal epithelial cell-specific deletion of *Dicer1* (Dicer^ΔIEC^) and analyzed intestinal phenotypes, including M-cell differentiation, morphology and function. Dicer^ΔIEC^ mice had a marked decrease in M cells compared to control floxed Dicer mice, suggesting an essential role of miRNAs in maturation of these cells. Furthermore, transmission electron microscopic analysis revealed that depletion of miRNA caused the loss of endosomal structures in M cells. In addition, antigen uptake by M cells was impaired in Dicer^ΔIEC^ mice. These results suggest that miRNAs play a significant role in M cell differentiation and help secure mucosal immune homeostasis.

## Introduction

The gastrointestinal tract is the site for digestion and absorption of nutrients, but at the same time it is exposed to foreign antigens including enormous numbers of commensal microorganisms as wells as pathogens. To protect from these foreign antigens, the gastrointestinal tract is equipped with a specialized gut-associated lymphoid tissue (GALT) as well as a variety of non-immunologic barriers, including gastric acid, pancreatic juice, bile, glycocalyx, a mucus layer, intercellular junctional complexes (e.g., tight junctions and adherens junctions), and rapid cell turnover [1, 2]. The mucosal surface is also protected by secretory antibody, especially immunoglobulin A, as well as antimicrobial peptides secreted from Paneth cells and enterocytes. In addition, GALT serves as the leading edge of an immunological barrier. GALT, comprised of Peyer’s patches (PPs), isolated lymphoid follicles, appendix and colonic patches is the main inductive site for mucosal immune responses [3]. Luminal surfaces of PPs are covered by the follicle-associated epithelium (FAE), which contains relatively limited numbers of goblet cells and enteroendocrine cells but harbors a unique subset of epithelial cells, membranous or microfold cells (M cells) [4]. Unlike the villus epithelium (VE), the FAE is specially designed to promote contact with luminal antigens to induce mucosal immune responses. For example, there are limited numbers of goblet cells in FAE and a thinner mucus layer compared to the VE region [4]. It has also been reported that FAE enterocytes lack polymeric Ig receptors for the local transport and secretion of secretory IgA [5]. In addition, antimicrobial peptide-producing Paneth cells are not present in the FAE crypts [6]. These features provide easier access to FAE by luminal particulate antigens such as bacteria and viruses. By contrast, the VE consists primarily of enterocytes, with scattered goblet cells and occasional enteroendocrine cells. The main function of the VE is the digestion and absorption of nutrients. Thus, the cellular composition and function of FAE and VE are quite different; however, the mechanisms that differentially regulate FAE and VE differentiation remain unknown.

M cells are specialized epithelial cells located in the FAE [7] that continuously sample and transport luminal antigens to the underlying GALT. The antigens are then captured by immature dendritic cells (DCs) residing in the subepithelial dome region beneath the FAE. The antigen-primed DCs undergo maturation and migrate to the T-cell area of GALT to present antigens to T cells, leading to activation of antigen-specific B cells and ultimately the production of IgA antibodies by lamina propria plasma cells [3]. Accumulated studies have begun to provide insight into antigen transcytosis and differentiation of M cells [8–12]; however, the molecular mechanisms underlying these processes are not fully elucidated.

MicroRNAs (miRNA) are ~19–25 nucleotide non-coding RNA molecules that regulate gene expression via repression of target mRNA. Binding of miRNAs to the 3' untranslated region of target mRNAs leads to translation inhibition or mRNA degradation [13]. A substantial number of studies have shown that miRNAs regulate many biological processes including cell or tissue development timing, differentiation and growth control [14]. The miRNAs are transcribed by RNA polymerase II as primary transcripts that are later processed by the RNase III-type endonuclease called Dicer into mature miRNAs. However, the complete loss of Dicer leads to embryonic lethality in mice [15], making its many functions difficult to study. To elucidate the importance of miRNAs in a particular tissue development or cell differentiation process, many groups have therefore used tissue or cell type-specific recombination approaches to deplete the *Dicer1* gene[16–19]. Expression profiles and functions of miRNAs in intestinal epithelium have been examined in jejunal and colonic mucosa [20], but those in FAE remain unknown. We generated intestinal epithelium-specific *Dicer1* deletion mice and investigated the role of miRNA in this tissue, focusing on the FAE. Here we report that miRNAs in FAE contribute to M-cell differentiation and that loss of miRNAs leads to impaired antigen transcytosis function via depletion of endosomes.

## Materials and Methods

### Animals

BALB/cA and C57BL/6 mice were purchased from CLEA Japan. *Dicer1* flox mice (Dicer^F/F^) were purchased from The Jackson laboratory and were backcrossed onto the C57BL/6 background. To generate intestinal epithelial cell-specific *Dicer1* knockout (Dicer^ΔIEC^) mice, we crossed *Dicer1* flox mice with villin-cre transgenic mice. Dicer^ΔIEC^ and control Dicer^F/F^ littermates were maintained under specific pathogen-free conditions. Mice are sacrificed by cervical dislocation. Animal experiments were approved by the Animal Research Committees of RIKEN and Yokohama City University [(Permit Numbers Kei 24–005 (RIKEN) and T11-001 (Yokohama City University)].

### Preparation FAE and VE for quantitative PCR

PPs were harvested from mice using curved scissors and flushed out the luminal contents with Hank’s balanced salt solution (HBSS). PPs were soaked in HBSS containing 30 mM EDTA for 20 min at 4°C. Epithelial cell sheets were peeled off from PPs, and FAE and VE were dissected by using 26G needles under stereomicroscopic monitoring ([21]) to obtain epithelium sheets of FAE and VE ([21, 22]). To count and calculate follicle numbers and surface area, PPs incubated with 3% acetic acid for 15 min at room temperature were analyzed using a SZX16 stereoscopic microscope (Olympus). Follicle surface area was calculated using DP2-BSW (Olympus).

### Quantitative PCR of miRNA

Total RNA including the small RNA fraction was extracted from murine FAE and VE using a mirVana kit (Ambion) and was reverse transcribed with a Taqman MicroRNA Reverse Transcription Kit (Applied Biosystems). Quantitative PCR was performed to quantify miRNA expression levels using the TaqMan Universal PCR Master Mix II w/ UNG (Applied Biosystems) and the Thermal Cycler Dice Real Time System (TAKARA). Values were normalized relative to the small nucleolar RNA *Sno202*. Specific primer sets were purchased from Applied Biosystems.

### Quantitative PCR of mRNA

Total RNA was extracted from murine FAE and VE using a mirVana kit (Ambion) and was reverse-transcribed using ReverTra Ace-α (TOYOBO). Quantitative PCR was performed to quantify mRNA expression levels using the SYBR Premix Ex Taq and the Thermal Cycler Dice Real Time System (TAKARA). Values were normalized to *Gapdh*. Specific primer pairs for each gene are listed in Table 1.

**Table 1:** Primer sequence of q-PCR.

Gene Symbol	Sequence
*Ccl9*	Forward 5’-TACTGCCCTCTCCTTCCTCA-3’
	Reverse 5’-TTGAAAGCCCATGTGAAACA-3'
*GAPDH*	Forward 5’-TGTGTCCGTCGTGGATCTGA-3’
	Reverse 5’-TTGCTGTTGAAGTCGCAGGAG-3'
*Gp2*	Forward 5’-GATACTGCACAGACCCCTCCA-3’
	Reverse 5’-GCAGTTCCGGTCATTGAGGTA-3'
*Marcksl1*	Forward 5’-TTTTGCCCTCCTGTGGATTCT-3’
	Reverse 5’-CCACTAGGCACAGCACAAGAGA-3'
*SpiB*	Forward 5’-AGCGCATGACGTATCAGAAGC-3’
	Reverse 5’-GGAATCCTATACACGGCACAGG-3'
*Dicer1*	Forward 5’-TGCCCTTGTCAATAACACCA-3’
	Reverse 5’-GCTCAGAGTCCATTCCTTGC-3'
*Mybl2*	Forward 5’-GGATGAAGATGGGAAGCTGA-3’
	Reverse 5’-TGAGCAGGCTGTTACCCTCT-3'

### Hematoxylin and Eosin (H&E) stain

Paraffin embedded tissue sections were deparaffinized and rehydrated. Then, sections were stained with hematoxylin and eosin.

### Whole mount immunostaining

PPs were excised from the small intestine, fixed with Cytofix/Cytoperm (BD Biosciences) for 1 hour at 4°C and then incubated with 10 μg/ml anti-CD16/32 monoclonal antibody (93; eBioscience) / 0.1% saponin / 0.2% BSA in phosphate buffered saline (PBS) to block non-specific Fc binding. The whole mount specimens were then stained overnight at 4°C with 1 μg/ml Alexa Fluor 488-conjugated anti-mouse GP2 (2F11C3; MBL) and 1 U/ml Alexa Fluor 555-conjugated Phalloidin (Molecular probes). The specimens were analyzed with a DM-IRE2 confocal laser scanning microscope and Leica confocal software (Leica Microsystems). To count M cells number, FAE sheet were peeled off from PPs as describe above. Then, FAE sheets were fixed with Cytofix/Cytoperm (BD Biosciences) for 1 hour at 4°C and incubated with 10 μg/ml anti-CD16/32 monoclonal antibody (93; eBioscience) / 0.1% saponin / 0.2% BSA in phosphate buffered saline (PBS) to block non-specific Fc binding. The whole mount specimens were then stained overnight at 4°C with 1 μg/ml Alexa Fluor 488-conjugated anti-mouse GP2 (2F11C3; MBL). The specimens were analyzed with a BX51 fluorescence microscope (Olympus). M cell count and FAE area measurement were examined using ImageJ software.

### Scanning electron microscopy

PPs were excised and fixed for 2.5 hours with 2.5% glutaraldehyde in 0.1 M phosphate buffer (pH7.4). The tissues were completely dehydrated in a graded ethanol series. Specimens were coated with a gold layer using a sputter coater MSP-1S (Shinku Device) and observed by SEM (VE-7800, KEYENCE).

### Transmission electron microscopy

PPs and VE were excised and fixed for 48 hours with 2.5% glutaraldehyde in 0.1 M phosphate buffer (pH7.4). The tissues were cut into 1–2 mm pieces, immersed for an additional 4 hours in the same fixative, postfixed for 1.5 hours with 1% OsO4 dissolved in distilled water, dehydrated in a graded series of ethanol, and embedded in Epon. Ultrathin sections were cut on an ultramicrotome and stained with uranyl acetate and lead citrate for observation under an electron microscope (H7100, Hitachi).

### Evaluation of bead uptake

8- to 10-week-old Diecr^F/F^ or Dicer^ΔIEC^ mice (four mice per group) were inoculated by gavage with 1 x 10^11^ FluoSpheres (Invitrogen). After 4 hours, PPs were dissected and incubated at 25°C in sterile PBS. Prepared frozen sections were examined using a BX51 fluorescence microscope (Olympus) and beads in PP follicles were counted manually.

### Evaluation of oral infection

8- to 10-week-old Diecr^F/F^ or Dicer^ΔIEC^ mice (five mice per group) were inoculated intragastrically by gavage with 0.2 ml of 0.1 M sodium bicarbonate to neutralize gastric acid. Mice were then inoculated intragastrically by gavage with 1 x 10^8^ CFU of *Yersinia enterocolitica* (ATCC27729). After 24 hours, three PPs from the ileal end were dissected and incubated at 25°C in sterile PBS containing 20 μg/ml gentamicin for 30 minutes, homogenized in sterile PBS and plated on Yersinia Selective Agar base (OXID) with Yersinia selective supplement (OXID) to determine CFU.

### Statistics

Statistical analysis was performed with the Mann-Whitney U test. Differences were considered as significant at *P* < 0.05.

## Results

### Different miRNA expression profiles in FAE and VE

**Fig 1:**
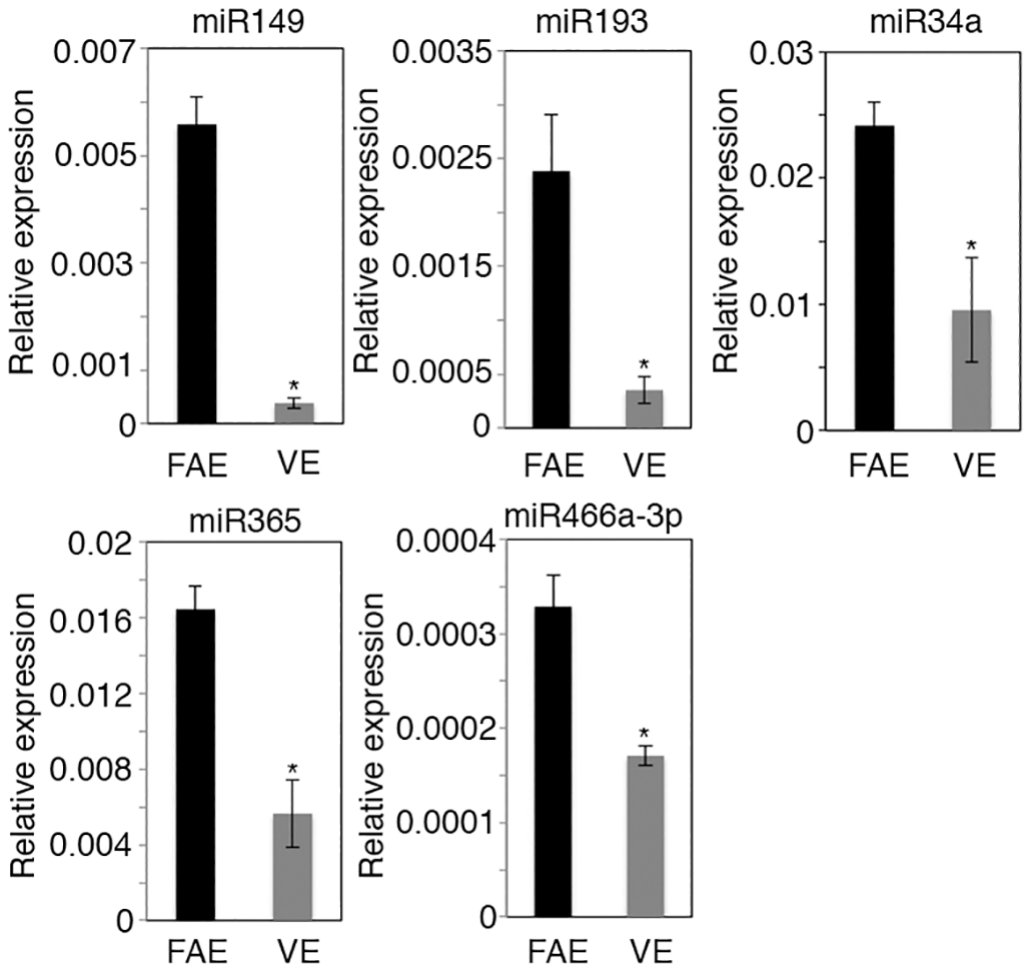
miRNA expression profiles in intestinal epithelium. Q-PCR analysis was performed for miRNA expression in FAE and VE. The relative levels of each miRNA relative to the small nucleolar RNA *Sno202* are shown. Values are mean ± SE of three samples from different mice. *P<0.05.

To examine the miRNA expression profile in FAE, we have previously dissected FAE and VE surrounding the FAE of PPs [11, 22] and compared their miRNA expression profiles by microarray analysis. We identified 43 miRNAs up-regulated at least two-fold in FAE compared with VE and 9 miRNAs down-regulated in FAE by at least two-fold [21]. The expression levels of the 43 miRNAs upregulated in FAE were further examined in C57BL/6 and BALB/cA mice by quantitative PCR (q-PCR). Only 5 miRNAs were commonly up-regulated at least two-fold in FAE compared with VE in both strains (Fig 1 and data not shown). We therefore reasoned that these five miRNAs are likely involved in FAE-specific translational regulation.

### miRNAs in FAE contribute to M-cell maturation

**Fig 2:**
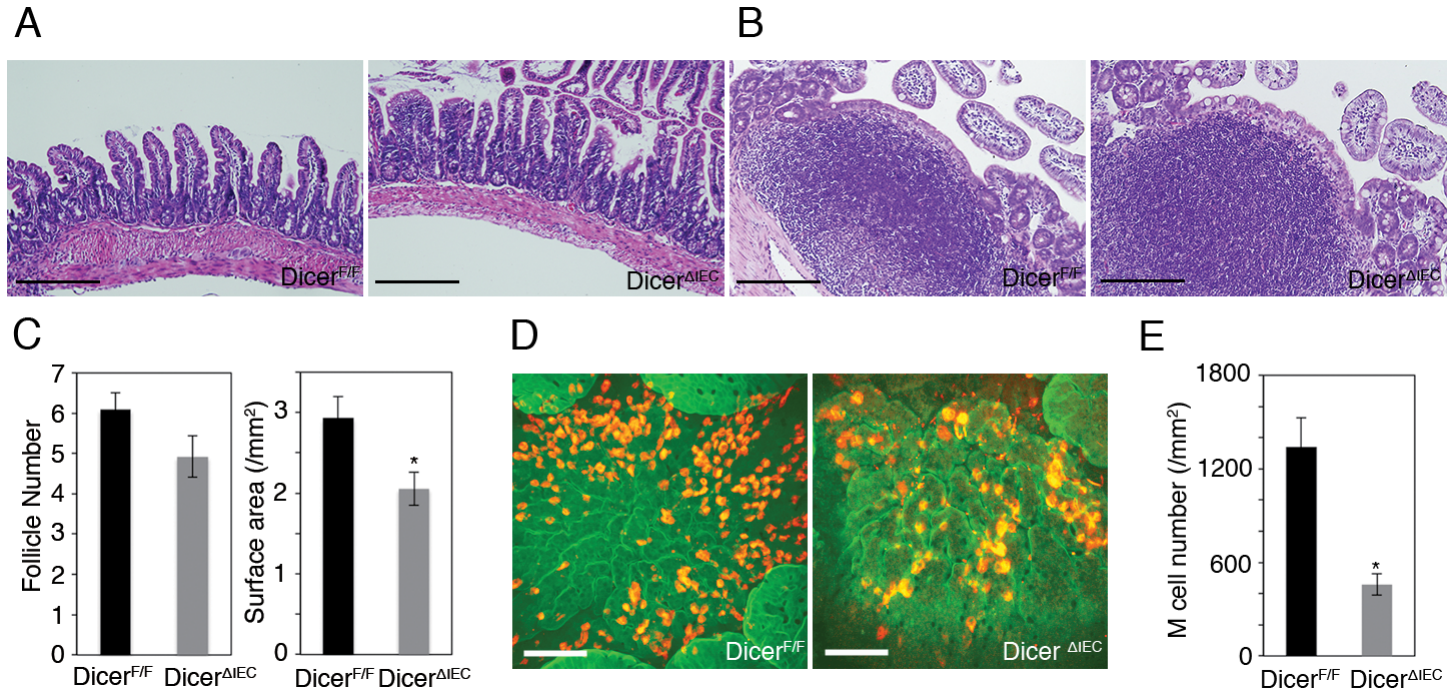
Total number of M cells in Peyer’s patches is decreased in Dicer^ΔIEC^ mice. (A) H&E staining of small intestines VE region of Dicer^ΔIEC^ and Dicer^F/F^. (B) H&E staining in PPs of Dicer^ΔIEC^ and Dicer^F/F^. (C) The total number of follicles and surface area in Dicer^ΔIEC^ and Dicer^F/F^. Data are means± SE (n = 3). *P < 0.05. (D) Whole mount immunostaining of PPs with anti-GP2 (red) and F-actin (green) analyzed using a confocal microscope. Scale bars: 100 μm (E) M cell number/mm^2^ in FAE of each mouse strain. Data are means and SE. *P < 0.05.

To elucidate the functions of miRNAs in intestinal epithelium, including FAE, we generated mice lacking mature miRNAs in intestinal epithelium by crossing floxed Dicer mice to villin-cre mice. Both Dicer^ΔIEC^ FAE and VE displayed approximately 85–95% reduction of Dicer1 mRNA expression levels compared with DicerF/F (S1 Fig) Dicer^ΔIEC^ mice showed decreased number of goblet cells and increased number of apoptotic cells in the crypt region. In addition, the number of Paneth and enteroendocrine cells was similar in both mice. These phenotypes are consistent with previous reports [20]. On the other hands, villous length and PPs structure in Dicer^ΔIEC^ were similar to Dicer^F/F^ (Fig 2A and 2B). Total number of PPs and follicles remained unchanged in Dicer^ΔIEC^ mice (Fig 2C, S2A and S2B Fig). By contrast, the surface area of FAE in Dicer^ΔIEC^ was less than that of control floxed Dicer (Dicer^F/F^) mice (Fig 2C, S2A and S2B Fig). In addition, whole mount immunostaining revealed that the density of M cells in FAE was decreased by more than half in Dicer^ΔIEC^ compared to Dicer^F/F^ mice (Fig 2D and 2E, S3 Fig).

**Fig 3:**
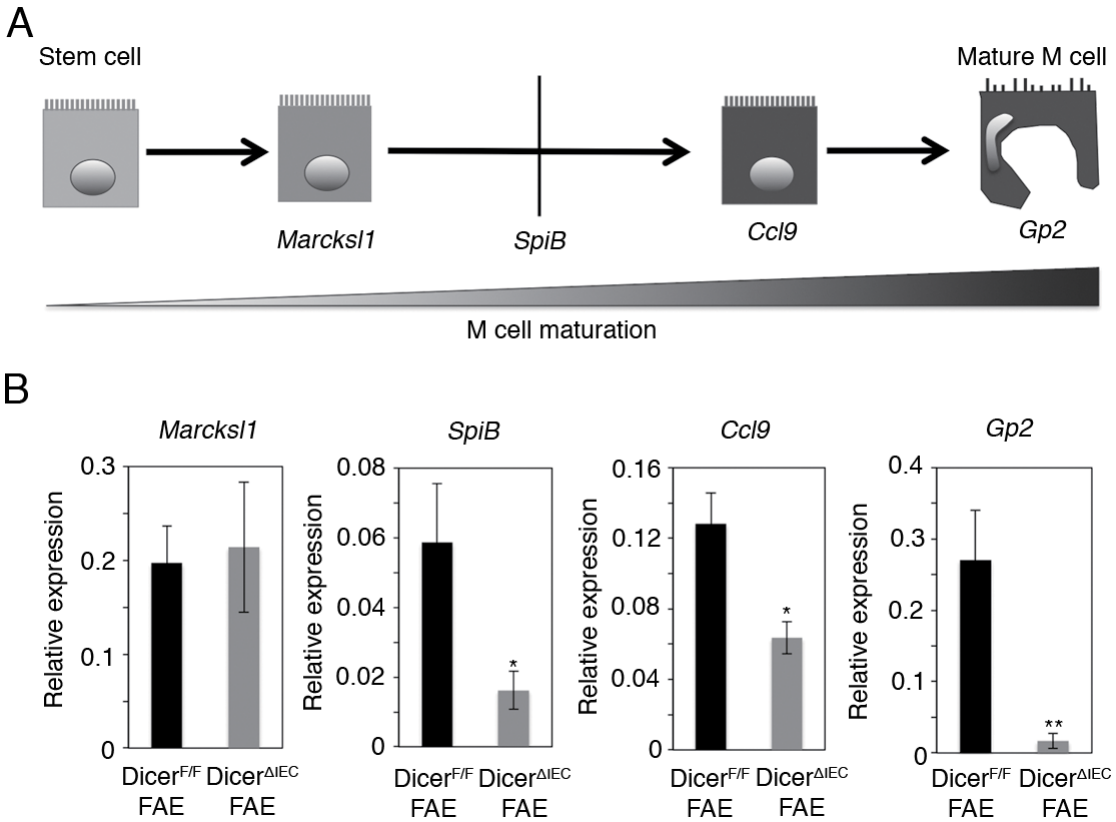
FAE miRNAs involved in M cell maturation. (A) Flowchart of M cell maturation. (B) Q-PCR analysis was performed for *Marcksl1*, *SpiB*, *Ccl9 and Gp2* mRNA expression in Dicer^ΔIEC^ FAE and Dicer^F/F^ FAE. The relative expression levels of each gene to *Gapdh* are shown. Values represent the mean ± SD of three samples from different mice. *P < 0.05 **P < 0.01.

**Fig 4:**
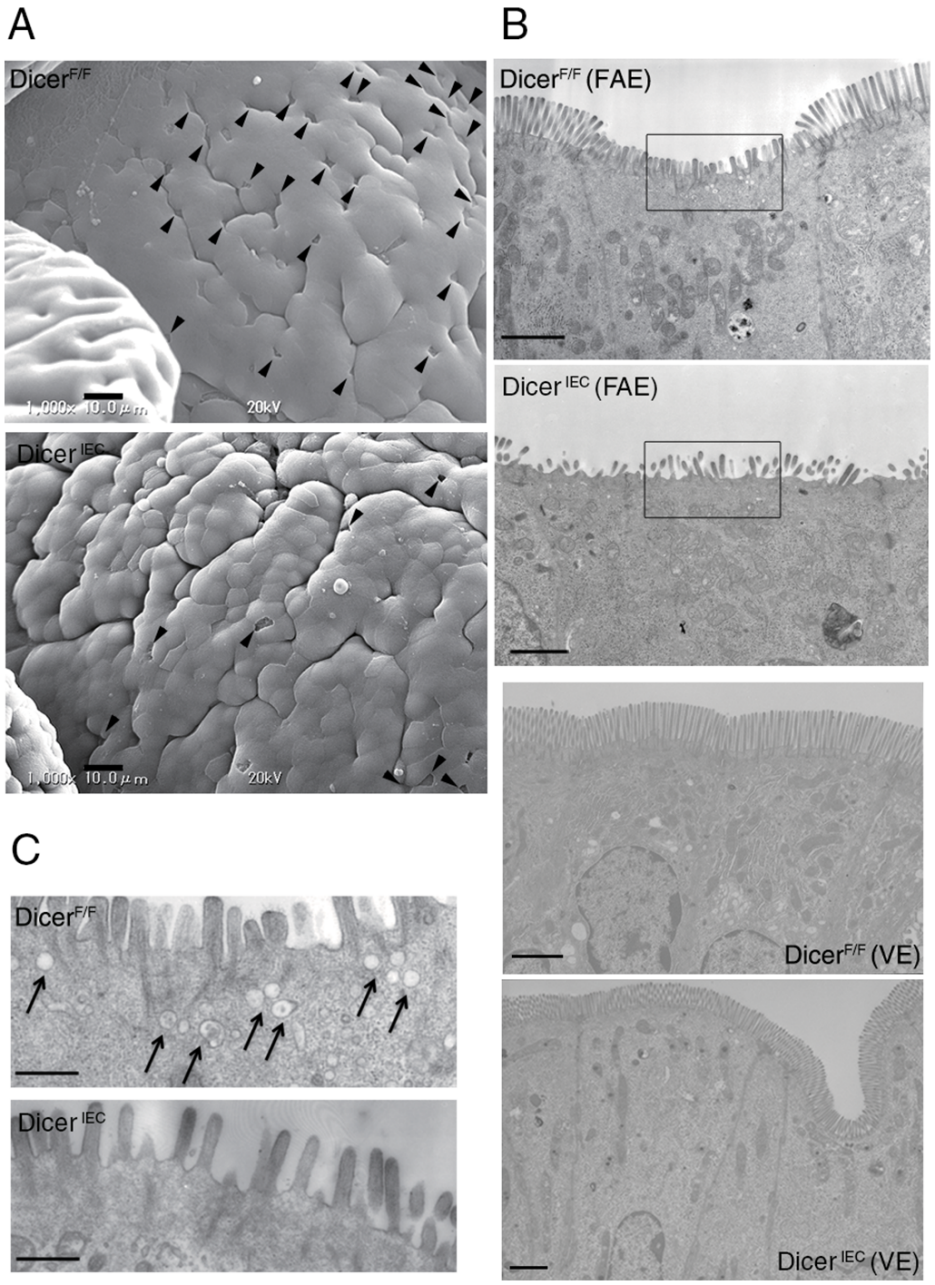
Morphology of M cells in Dicer^ΔIEC^. Electron micrographs of Dicer^F/F^ and Dicer^ΔIEC^ PP. (A) Surface of FAE by scanning electron microscopy. Arrowheads indicate M cells. Scale bars: 10 μm (B) Transmission electron micrographs of an M cell in FAE and enterocyte in VE. Scale bars: 1.8 μm (C) High magnification image of (B). Arrow indicate the endosomes in M cell. Scale bars: 500 nm.

Recent studies suggests that maturing M cells can be classified into distinct differentiation stages based on the expression of M-cell markers (Fig 3A and [10, 23]), therefore, we next examined their expression by q-PCR. The mRNA levels of *SpiB*, *Ccl9* and *Gp2* were significantly decreased in Dicer^ΔIEC^ FAE compared to Dicer^F/F^; however, expression of the earliest known M-cell marker *Marcksl1* was the same in both mice (Fig 3B). We also examined the morphology of M cells by electron microscopy. Scanning electron micrographs of FAE showed a decrease in the number of cells with the typical M-cell morphology, i.e. organized microvilli on the apical surface, in Dicer^ΔIEC^ FAE (Fig 4A). To understand in more detail the M-cell morphology, we also performed transmission electron microscopic (TEM) analysis, confirming the loss of organized microvilli (Fig 4B). On the other hand, morphology of microvilli in Dicer^ΔIEC^ VE was similar to that in Dicer^F/F^ (Fig 4B). These results are consistent with whole mount immunostaining. Taken together, these results indicate that miRNAs in FAE are important for M-cell maturation.

### Impaired antigen uptake by Dicer^ΔIEC^ M cells

Many endocytic/endosomal structures were observed by TEM in the Dicer^F/F^ M cells, whereas these structures were nearly absent in the Dicer^ΔIEC^ M cells (Fig 4B and 4C), suggesting that endocytic activity is impaired in Dicer^ΔIEC^ M cells.

**Fig 5:**
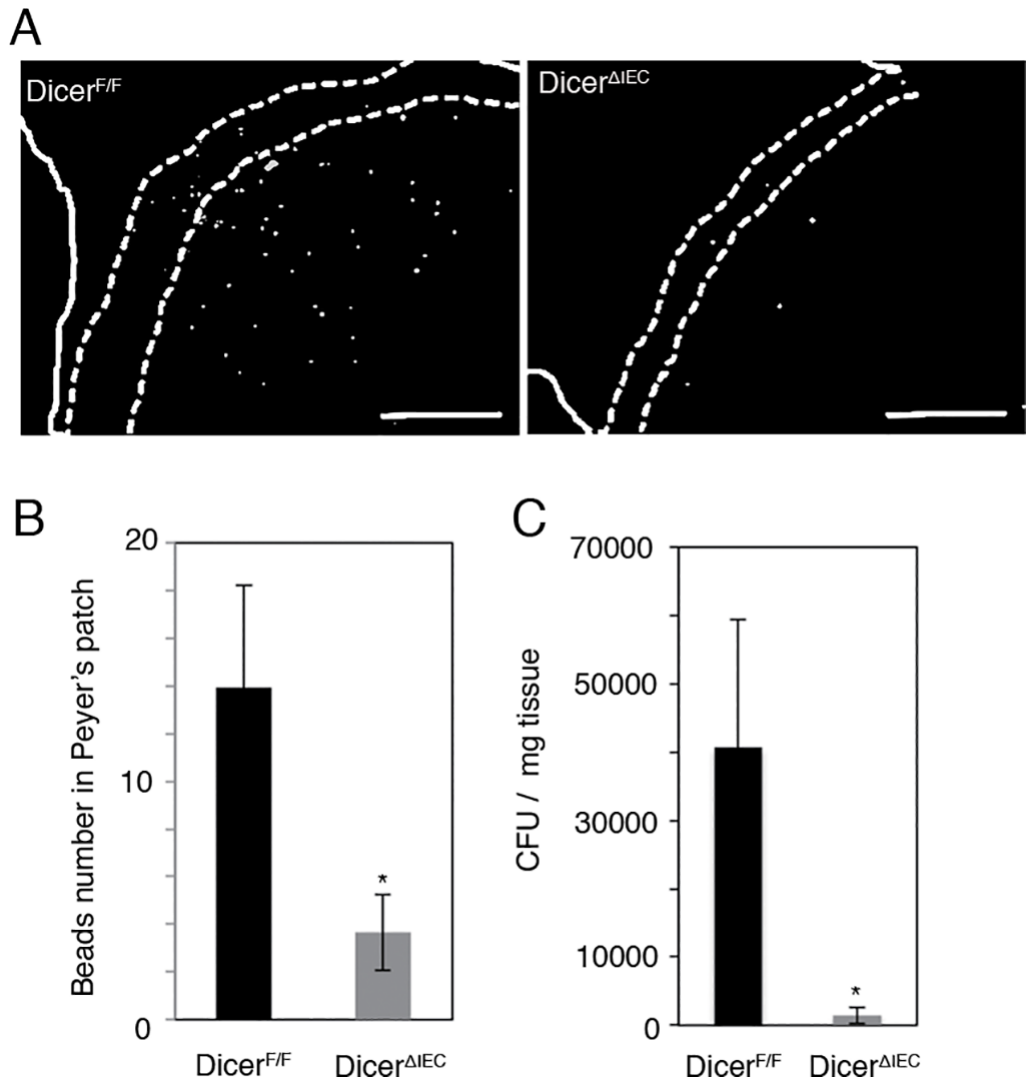
Impaired antigen uptake by Dicer^ΔIEC^ M cells. (A) Dicer^ΔIEC^ and Dicer^F/F^ mice were inoculated by gavage with 1 x 10^11^ FluoSpheres. After 4 hours, frozen sections were prepared to examine translocated beads in PPs. Scale bars: 100 μm (B) Count data of beads taken up in PP each mouse strain. Data are expressed as the mean ± SE of four different samples for each group. **P < 0.01. (C) Dicer^ΔIEC^ and Dicer^F/F^ mice were inoculated intragastrically by gavage with 1 x 10^8^ CFU of *Yersinia enterocolitica*. After 24 hours, the bacterial translocation to Peyer’s patches was examined by plating PP homogenates. Data are expressed as the mean ± SE of five different mice/each group. *P < 0.05.

To verify this point, we inoculated mice with fluorescent beads via the oral route. The beads were easily detected in the subepithelial dome region of Dicer^F/F^ PPs, whereas very few were observed in Dicer^ΔIEC^ PPs (Fig 5A and 5B). We further investigated the impaired antigen uptake by M cells in the PPs of Dicer^ΔIEC^ mice by examining the translocation of orally administered *Yersinia enterocolitica* and found that it was markedly reduced in Dicer^ΔIEC^ compared to Dicer^F/F^ mice (Fig 5C). Taken together, our results indicate that FAE miRNA is important for both morphological and functional maturation of M cells.

## Discussion

To ensure efficient antigen uptake, the cellular composition and function of FAE differs significantly from VE. Besides absorptive enterocytes, the VE is populated with scattered goblet cells and enteroendocrine cells, while the FAE consists of very few goblet and enteroendocrine cells and instead contains M cells [4]. We identified five miRNAs that are two-fold or more upregulated in FAE compared to VE in C57BL/6 (Fig 1) and BALB/cA mice (data not shown), thus it seemed likely that miRNAs may affect or regulate the cell distribution or function in FAE.

To test this possibility, we generated intestinal epithelium-specific Dicer-KO (Dicer^ΔIEC^) mice. A similar strategy has been taken by others, who have reported that miRNAs are involved in goblet-cell differentiation in the intestine [20], supporting the notion that this is a reliable approach to examine the role of miRNAs in intestinal epithelial cells. The total number of PPs as well as lymphoid follicles remained unchanged, but the size of the FAE in Dicer^ΔIEC^ mice was smaller than in Dicer^F/F^ mice (Fig 2C). In addition, the total number of immune cells in PPs also decreased in Dicer^ΔIEC^ mice (data not shown). Chemokines specifically expressed by the FAE are thought to be important for recruitment of immune cells to PPs [8, 24]. For example, CXCL16 expressed by the FAE plays a critical role in the recruitment and retention of T cells in the subepithelial dome [24], while CCL20 derived from FAE is important for migration of CCR6^hi^CD11c^int^ B cells [8]. Furthermore, CCL20 is also a crucial chemokine for M-cell differentiation and/or maintenance, since mice lacking CCR6, the sole receptor for CCL20, had a reduction in M cells [25, 26]. Of note, *Ccl20* and *Cxcl16* mRNA was decreased in Dicer^ΔIEC^ FAE compared to Dicer^F/F^ (data not shown). Collectively, the phenotype of Dicer^ΔIEC^ PPs described in this study may at least partly reflect the dysregulated expression of these chemokines by intestinal epithelial miRNAs.

Dicer^ΔIEC^ mice had a prominent decrease in the number of mature M cells (Fig 2D and 2E, S3 Fig). The M-cell reduction was confirmed by electron microscopy (Fig 4A). Concomitantly, one of the main functions of M cells, mucosal antigen transcytosis, was impaired in Dicer^ΔIEC^ mice PP, as measured by uptake of beads and by bacterial translocation (Fig 5A, 5B and 5C). In addition, the small number of M cells remaining in Dicer^ΔIEC^ FAE lack endocytic/endosomal structures normally observed (Fig 4B and 4C). These results indicate that FAE miRNAs are important for differentiation and/or maturation of functional M cells.

In this study, we identified five miRNAs that were up-regulated at least two-fold in FAE compared with VE in both C57BL/6 and BALB/cA mice (Fig 1). Among them, two miRNAs, miR34a and miR365, might be involved in M-cell maturation. The miR34a is a member of the miR-34 family, which is conserved from *C*. *elegans* to mammals. Recent studies have shown that miR-34a regulates the notch signaling pathway [27], for example, miR-34a represses Delta-like 1 (Dll1) [28] and miR34a down-regulation leads to increased Notch1 and Jag1 expression [29]. We showed in this study that the expression of M-cell marker genes such as *SpiB*, *Ccl9* and *Gp2* was much less in Dicer^ΔIEC^ mice than in Dicer^F/F^ mice (Fig 3B). The Ets family transcription factor Spi-B plays a critical role in M-cell differentiation [10]. Dicer^ΔIEC^ mice showed a phenotype similar to the *Spib*-knockout mice [10], which raises the possibility that miRNAs regulate *Spib* expression. It has been reported that Dll1-induced Notch1 signaling induces down-regulation of Spi-B in plasmacytoid DCs [30]. Taken together with our results, it seems possible that miR34a also regulates *Dll1*, *Jag1* and *Notch1* in FAE and this might, in turn, promote Spi-B expression required for M-cell maturation. Because miR34a was highly expressed in FAE among miRNAs examined, we tried to generate miR34a null mice for the examination of M cells development. However, miR34a heterozygous crossing could not give rise to any miRNA34a null mice, The result may indicate that miRNA34a plays a crucial role in murine embryonic development, and precludes the possibility for us to use these mice to study the role miR34a in M-cell/FAE development.

During colonic epithelium maturation, miR365 has been reported to regulate Myb-related protein B (MYBL2) expression, which is important in both cell cycle regulation and differentiation in various biological processes [31]. Reduction of MYBL2 expression is important for acquisition of full differentiation of colonic epithelial cell lines [32]. However *Mybl2* mRNA expression was similar expression levels between Dicer^ΔIEC^ FAE and Dicer^F/F^ (S4 Fig). Therefore, the *Mybl2* expression level controlled by miR365 may not involve in M-cell maturation. Functions of the other three miRNAs, miR149, miR193 and miR466a-3p, have not been previously examined in the intestinal epithelium *in vitro* or *in vivo*. Generation of individual or multiple miRNA null or individual miRNA conditional knockout mice will help to further understand the mechanisms of M-cell maturational regulation by these miRNAs.

In conclusion, our data suggest that intestinal epithelial miRNAs are critical for the morphological and functional maturation of M cells. The appropriate regulation of gene expression by intestinal epithelial miRNAs should contribute to the cell fate decision and/or maintenance of cellular differentiation and functions in mucosal immune homeostasis.

## Supporting Information

S1 Fig *Dicer1* mRNA dramatically decrease Dicer^ΔIEC^ epithelium. Q-PCR analysis was performed for *Dicer1* mRNA expression in FAE and VE in Dicer^ΔIEC^ and Dicer^F/F^. The relative expression levels of each gene to *Gapdh* are shown. Values represent the mean ± SD of three samples from different mice. *P < 0.05.
(TIF)

S2 Fig Compare follicle number between Dicer^ΔIEC^ and Dicer^F/F^. Stereomicroscopic images of Dicer^F/F^ PPs (A) and Dicer^ΔIEC^ PPs (B) after citric acid fixation. Asterisk showed individual follicle. Solid line in (A) showed representative area of calculated follicle surface. Scale bars: 500 μm.
(TIF)

S3 Fig GP2 positive M cell decreased in FAE. Whole mount immunostaining of isolated epithelial sheet with anti-GP2 (Green) analyzed using BX51 fluorescence microscope (Olympus). Solid line showed FAE region. Scale bars: 200 μm.
(TIF)

S4 Fig *Mybl2* levels was similar between Dicer^F/F^ FAE and Dicer^ΔIEC^ FAE. Q-PCR analysis was performed for *Mybl2* mRNA expression in Dicer^ΔIEC^ FAE and Dicer^F/F^ FAE. The relative expression levels of each gene to *Gapdh* are shown. Values represent the mean ± SD of three samples from different mice.
(TIF)

## Abbreviations

GALT: gut-associated lymphoid tissue
PPs: Peyer’s patches
FAE: Follicle associated epithelium
M cells: microfold cells
VE: villus epithelium
DC: dendritic cell
miRNA: micro RNA
PBS: Phosphate buffered saline
Dll1: delta-like 1
