# Supplementary figures and images for: Epithelium-Intrinsic MicroRNAs Contribute to Mucosal Immune Homeostasis by Promoting M-Cell Maturation
Source: PLoS One. 2016 Mar 1;11(3):e0150379. doi: 10.1371/journal.pone.0150379 (PMC4773159; doi:10.1371/journal.pone.0150379)

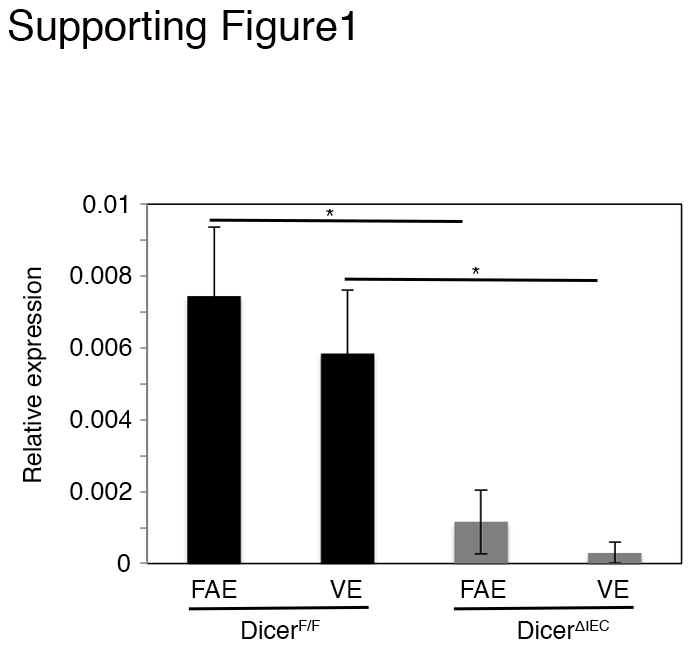

Supplement: S1 Fig — Q-PCR analysis was performed for Dicer1 mRNA expression in FAE and VE in DicerΔIEC and DicerF/F. The relative expression levels of each gene to Gapdh are shown. Values represent the mean ± SD of three samples from different mice. *P < 0.05. (TIF) [file pone.0150379.s001.tif]

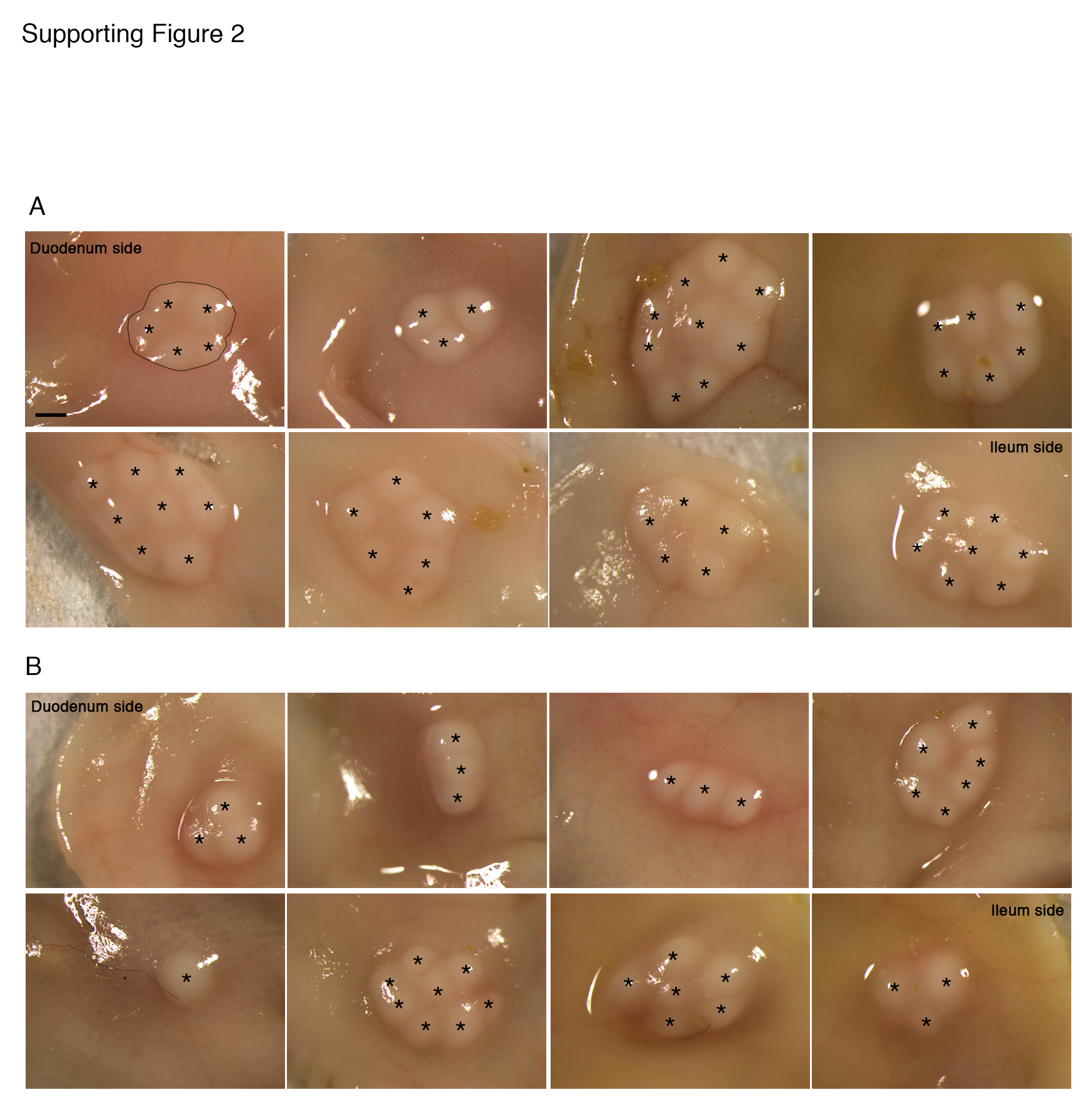

Supplement: S2 Fig — Stereomicroscopic images of DicerF/F PPs (A) and DicerΔIEC PPs (B) after citric acid fixation. Asterisk showed individual follicle. Solid line in (A) showed representative area of calculated follicle surface. Scale bars: 500 μm. (TIF) [file pone.0150379.s002.tif]

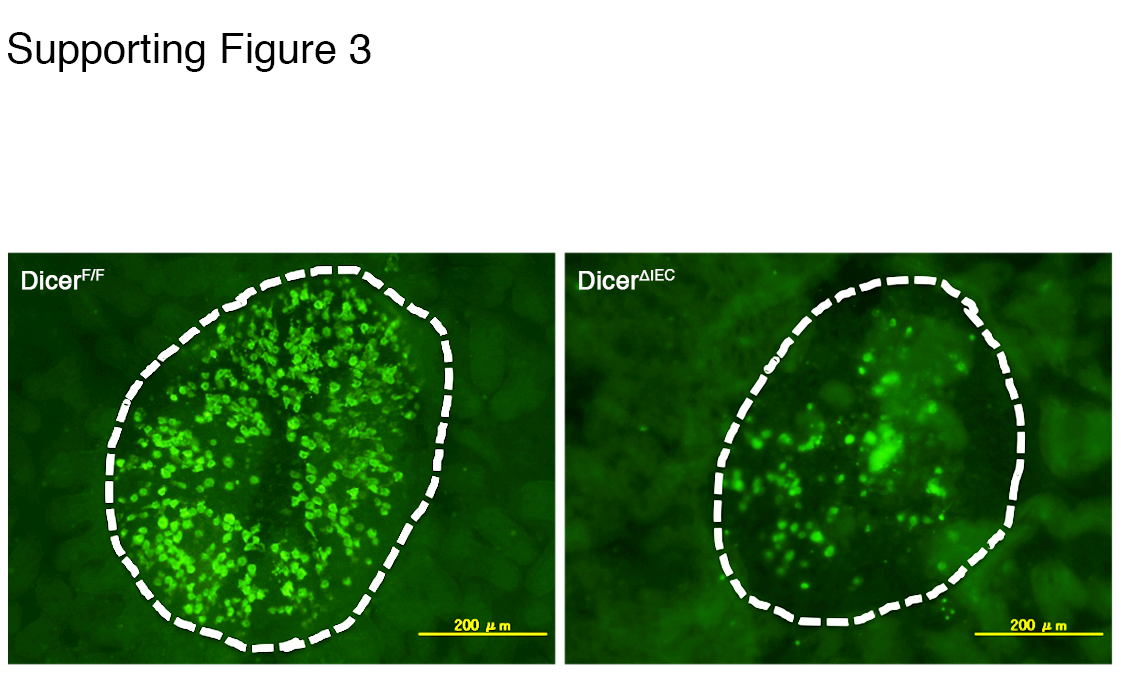

Supplement: S3 Fig — Whole mount immunostaining of isolated epithelial sheet with anti-GP2 (Green) analyzed using BX51 fluorescence microscope (Olympus). Solid line showed FAE region. Scale bars: 200 μm. (TIF) [file pone.0150379.s003.tif]

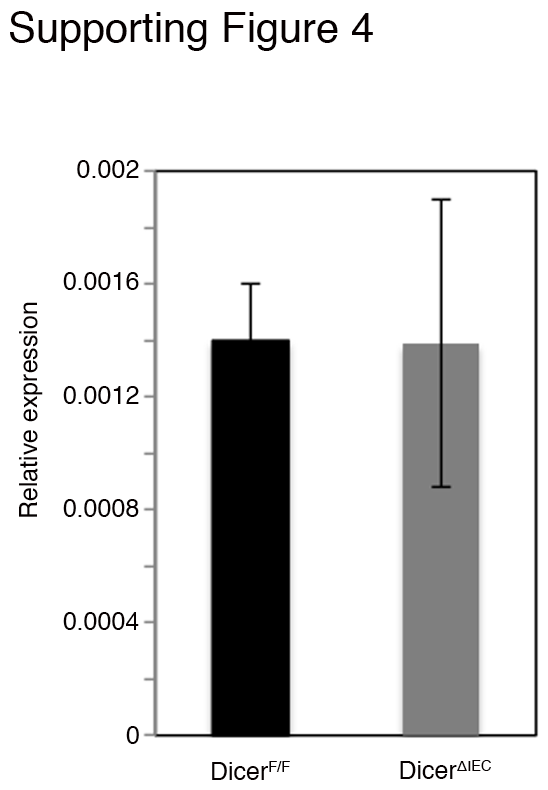

Supplement: S4 Fig — Q-PCR analysis was performed for Mybl2 mRNA expression in DicerΔIEC FAE and DicerF/F FAE. The relative expression levels of each gene to Gapdh are shown. Values represent the mean ± SD of three samples from different mice. (TIF) [file pone.0150379.s004.tif]
